# 靶向CD33抗原三特异性T细胞衔接器的制备及对白血病细胞的作用研究

**DOI:** 10.3760/cma.j.issn.0253-2727.2022.05.005

**Published:** 2022-05

**Authors:** 婷 张, 曼玲 陈, 晓雨 刘, 慧珍 何, 颖茜 徐, 征 田, 海燕 邢, 克晶 唐, 青 饶, 敏 王, 建祥 王

**Affiliations:** 中国医学科学院北京协和医学院血液病医院（中国医学科学院血液学研究所），实验血液学国家重点实验室，国家血液系统疾病临床医学研究中心，细胞生态海河实验室，天津市血液病细胞治疗研究重点实验室，天津 300020 State Key Laboratory of Experimental Hematology, National Clinical Research Center for Blood Diseases, Tianjin Key Laboratory of Cell Therapy for Blood Diseases, Haihe Laboratory of Cell Ecosystem, Institute of Hematology & Blood Diseases Hospital, Chinese Academy of Medical Sciences & Peking Union Medical College，Tianjin 300020, China

**Keywords:** 白血病，髓样，急性, 三特异性T细胞衔接器, 免疫治疗, Leukemia, myeloid, acute, Trispecific-T cell Engagers, Immunotherapy

## Abstract

**目的:**

研究靶向CD33抗原双特异性及三特异性T细胞衔接器对T细胞增殖及其抗白血病作用。

**方法:**

构建抗CD33scFv-抗CD3scFv的双特异性T细胞衔接器（CD33-BiTE）及在BiTE基础上加入CD80胞外段的三特异性T细胞衔接器（CD33-TriTE）表达载体，使用真核细胞表达系统表达蛋白并进行亲和层析纯化。检测CD33-BiTE及CD33-TriTE对T细胞活化增殖功能及对白血病细胞杀伤功能活性的影响。

**结果:**

①成功构建了CD33-BiTE及CD33-TriTE表达载体，在真核细胞中表达，纯化得到的融合蛋白能与相同靶抗原流式抗体竞争结合于靶细胞表面。②CD33-BiTE及CD33-TriTE分别与人T细胞共培养12 d后，T细胞数扩增至基线值的（33.89±19.46）倍和（81.54±23.62）倍，CD33-TriTE促T细胞增殖能力明显优于CD33-BiTE（*P*<0.05）。③体外实验证实CD33-BiTE和CD33-TriTE均可增强T细胞对表达CD33白血病细胞的特异性杀伤作用，且在一定浓度范围内，浓度越高，抗体的杀伤作用越强。④与CD33-TriTE相比，CD33-BiTE杀伤白血病细胞的同时增加其PD-L1表达，而TriTE对过表达PD-L1的Molm13细胞具有更强的杀伤作用。

**结论:**

该研究构建了CD33-BiTE及CD33-TriTE表达载体，并在真核细胞表达了融合蛋白，体外实验证实其促T细胞增殖活化的特性，并具有促T细胞抗白血病作用。其中CD33-TriTE较CD33-BiTE促增殖效果更强，且对PD-L1高表达的白血病细胞杀伤效果更强。

## Abstract (en)

**Objective:**

To investigate the effect of CD33-targeted bi-specific and tri-specific T-cell engagers on T-cell proliferation and explore their cytotoxicity on leukemia cells.

**Methods:**

The CD33-targeted bi-specific T-cell engager（CD33-BiTE）and tri-specific T-cell engager（CD33-TriTE）expression vectors were successfully constructed and expressed through a eukaryotic cell expression system. CD33-BiTE and CD33-TriTE were purified by affinity chromatography. The effects of CD33-BiTE and CD33-TriTE on T cells were analyzed through in vitro experiments.

**Results:**

① CD33-BiTE and CD33-TriTE were successfully constructed and purified and could compete with flow cytometry antibodies for binding to the target cells. ② After 12 days of co-culture with CD33-BiTE and CD33-TriTE, the number of human T cells were expanded to 33.89±19.46 and 81.56±23.62 folds, respectively. CD33-TriTE induced a stronger proliferation of T cells than CD33-BiTE（*P*<0.05）. ③ Both CD33-BiTE and CD33-TriTE induced specific dose-dependent cytotoxicity on CD33^+^ leukemia cells. ④ Compared to CD33-TriTE, leukemia cells were prone to express PD-L1 when co-cultured with T cells and CD33-BiTE. CD33-TriTE induced powerful cytotoxicity on leukemia cells with high PD-L1 expression.

**Conclusion:**

CD33-BiTE and CD33-TriTE expression vectors were constructed, and fusion proteins were expressed in eukaryotic cells. Our results support the proliferative and activating effects of BiTE and TriTE on T cells. Compared to that of CD33-BiTE, CD33-TriTE induced a stronger proliferative effect on T cells and a more powerful cytotoxicity on leukemia cells with high PD-L1 expression.

急性髓系白血病（AML）治疗方案由基于柔红霉素或去甲氧柔红霉素及阿糖胞苷为基础的“7+3”化疗方案及后续巩固治疗及造血干细胞移植组成，60％～80％年轻患者和40％～60％的老年患者能得到缓解[1]，但仍有部分患者出现难治或复发[2]。近年来，免疫治疗如嵌合抗原受体T细胞（CAR-T细胞）被应用于AML的治疗中[3]；针对白血病细胞上CD33的高表达，靶向CD33的抗体偶联药物已被应用于临床一线治疗[4]；同时，各种靶向CD33的双特异性T细胞衔接器（BiTE）也正在进行相关临床前及临床研究[5]–[8]。本研究中，我们开发了一种靶向CD33的BiTE，并在BiTE的基础上加入CD80胞外段，构成三特异性T细胞衔接器（TriTE），对BiTE及TriTE功能活性进行了比较。

## 材料与方法

一、主要材料、试剂

ExpiCHO-S细胞、ExpiCHO瞬时转染试剂购于美国Thermo Fisher公司；His Tag蛋白纯化柱购于美国GE公司；蛋白脱盐及浓缩柱购于美国Milllipore公司；蛋白定量His-ELISA检测试剂盒购于南京金斯瑞生物科技股份有限公司；淋巴细胞培养液购于美国Corning公司；RPMI 1640培养基购于美国Gibco公司；胎牛血清购于法国Biowest公司；重组人IL-2因子购于美国R&D公司；EndoFree Plasmid Maxi质粒抽提试剂盒购自美国Invitrogen公司；ONE-Glo Luciferase活性检测试剂盒购自美国Promega公司。所有流式一抗均购于美国Biolegend公司；RossetteSep T细胞富集试剂购于美国Stem Cell公司。

二、细胞株培养

人AML细胞系Molm13细胞、过表达Luciferase基因的Molm13-luc2细胞、Jurkat细胞、Namalwa细胞培养于含10％胎牛血清RPMI 1640培养液；人T细胞用Ficoll淋巴细胞分离液及RossetteSep T细胞富集试剂富集后，培养于含10％胎牛血清、100 IU/ml重组人IL-2因子培养液中。

三、载体构建及蛋白表达

CD33单链可变区片段（scFv）及CD3 scFv为本实验室前期构建的知识产权序列[9]–[10]。CD80胞外段表达区序列从NCBI查询获取。将CD33 scFv-CD3 scFv或CD33 scFv-CD80-CD3 scFv克隆到pcDNA3.4质粒，并送测序鉴定。参照ExpiCHO瞬时转染表达系统手册转染ExpiCHO细胞，转染第14天收取细胞培养基，1 962×*g*离心30 min取上清液。Western blot验证蛋白表达后，用0.45 mm滤膜过滤后进行标签为His tag的镍柱纯化，使用不同浓度的咪唑进行漂洗和洗脱。纯化后蛋白使用Millipore超滤浓缩柱进行脱盐及浓缩，使用His-ELISA试剂盒定量。

四、竞争结合活性检测

将等浓度CD33-BiTE与CD33-TriTE分别加入表达CD33的Molm13细胞系及表达CD3的Jurkat细胞系共孵育30 min，而后加入CD33或CD3流式抗体竞争结合共孵育30 min，流式细胞术检测靶抗原荧光强度下降情况。

五、对人T细胞活化增殖作用

1. T细胞增殖绝对值计数：用0.1 nmol/L的CD33-BiTE或CD33-TriTE蛋白与T细胞共孵育，加入50 U 人IL-2，对照组加入等体积PBS。在37 °C，5％CO_2_孵箱中培养，从第4天起每隔1天使用细胞计数仪计数，计算T细胞增殖情况。

2. T细胞活化及分群检测：以上条件培养T细胞时，取第4天细胞上流式细胞仪检测CD8^+^ T细胞CD25表达代表活化情况。取第8天细胞上流式细胞仪检测CD8^+^T细胞比例。

六、介导人T细胞对白血病细胞的杀伤作用

1. 流式细胞术检测AML细胞系及患者原代AML细胞中CD33的表达情况：用FITC标记的抗CD33单抗标记Molm13细胞、Namalwa细胞，检测CD33的表达水平。

2. 流式细胞术检测BiTE及TriTE细胞的体外杀伤活性：在0.1 nmol/L的BiTE或TriTE培养基体系里，人T细胞与Molm13细胞、Namalwa细胞按效靶比2∶1共培养24、60 h，标记CD33或CD22、CD3流式抗体并检测CD33^+^靶细胞及CD3^+^ T细胞比例，记录剩余靶细胞比例为不同时间CD33^+^靶细胞与0 h起始CD33^+^靶细胞比例。

3. 萤火虫萤光素酶化学发光发检测BiTE及TriTE细胞的体外杀伤活性：在不同浓度的BiTE或TriTE培养基体系里，人T细胞与实验室建立的携带萤火虫Molm13-luc2细胞系按效靶比2∶1共培养72 h后，加入100 ml ONE-Glo Luciferase活性检测试剂，室温孵育10 min后使用酶标仪检测并计算杀伤活性。

杀伤活性＝（杀伤率_样品孔_−杀伤率_基线孔_）/（1−杀伤率_基线孔_）×100％

4. 脱颗粒实验分析T细胞被BiTE及TriTE激活：在0.1 nmol/L的BiTE或TriTE培养基体系里，人T细胞与Molm13细胞、Namalwa细胞按效靶比2∶1共培养5 h后（同时加入莫能霉素抑制蛋白转运），流式细胞仪检测CD8^+^ T细胞CD107a表达比例，反映细胞激活比例。

七、构建过表达PD-L1的Molm13细胞系

PD-L1序列来源于NCBI查询获取，将合成的PD-L1序列克隆至慢病毒载体中。应用Lipo3000脂质体转染法分别将PD-L1慢病毒载体质粒、Gag-pol质粒、VSV-G质粒共同转入293T包装细胞系，培养24～72 h收集病毒上清。慢病毒上清经离心沉淀后加入Molm13细胞系，同时添加Polybrene 6 µg/ml，706×*g* 31 °C离心1.5 h促进转染。转染6 h后换液，流式抗体标记PD-L1并测定表达。

八、统计学处理

采用GraphPad Prism 8.0.2软件进行统计学分析。实验重复3次，连续型变量以均值±标准差表示，组间差异分析采用单因素方差分析，组内两两比较采用SNK检验。*P*<0.05为差异有统计学意义。

## 结果

1. BiTE及TriTE结构设计及亲和力结合特异性鉴定：将CD33scFv-CD80胞外表达区-CD3scFv基因序列依次串联连接，重组蛋白中的不同基因序列用柔性连接肽相连，成功构建pcDNA3.4-TriTE表达载体。将CD33scFv-CD3scFv基因序列以柔性连接肽相连，成功构建pcDNA3.4-BiTE表达载体（图1A），Linker1为（G4S）_3_，Linker2为（G4S）_4_。经过真核细胞表达、纯化后获得与His抗体结合的条带，CD33-BiTE与CD33-TriTE在60×10^3^及110×10^3^附近有主条带，与预期相符（图1B）。

**图1:**
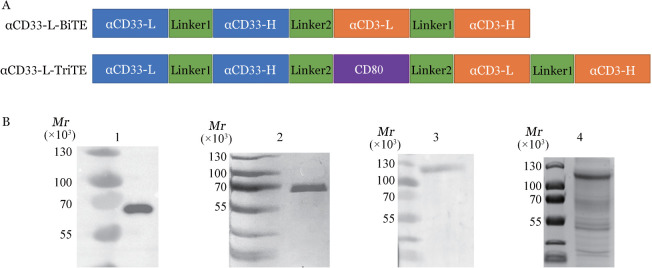
CD33-BiTE及CD33-TriTE结构设计及表达鉴定 BiTE：双特异性T细胞衔接器；TriTE：三特异性T细胞衔接器；A：CD33-BiTE及CD33-TriTE结构示意图；B：考马斯亮蓝及Western Blot鉴定CD33-BiTE及CD33-TriTE蛋白表达（1：CD33-BiTE/Western Blot；2：CD33-BiTE/考马斯亮蓝；3：CD33-TriTE/Western Blot；4：CD33-TriTE/考马斯亮蓝）

将CD33-BiTE与CD33-TriTE分别加入表达CD33的Molm13细胞系及表达CD3的Jurkat细胞共孵育，而后加入CD33或CD3流式抗体竞争结合，可见CD33-BiTE与CD33-TriTE均能封闭CD33或CD3的抗原抗体结合位点（图2A）。表明CD33-BiTE与CD33-TriTE均有与CD33和CD3的抗体亲和结合能力。将CD33-BiTE、CD33-TriTE或PBS与Molm13或T细胞共孵育后，CD33-TriTE组CD80阳性细胞比例增加，考虑为CD33-TriTE结合于Molm13及T细胞表面的CD80胞外段（图2B）。

**图2:**
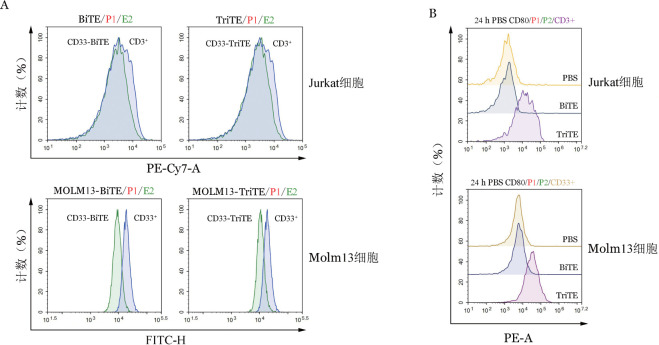
CD33-BiTE与CD33-TriTE竞争结合活性鉴定（A）及CD80表达鉴定（B） BiTE：双特异性T细胞衔接器；TriTE：三特异性T细胞衔接器

2. BiTE及TriTE对T细胞增殖与活化影响：将人T细胞与CD33-BiTE及CD33-TriTE共培养后，对T细胞进行绝对值计数。与PBS组相比，BiTE与TriTE组从第4天起细胞数量明显增加，至第12天细胞计数增至基线值的（33.89±19.46）倍和（81.54±23.62）倍，CD33-TriTE促T细胞增殖能力明显高于CD33-BiTE（*P*<0.05）（图3A）。培养第4天检测CD8^+^ T细胞CD25表达情况，PBS组、BiTE组、TriTE组中CD8^+^ T细胞中CD25^+^细胞比例分别为（5.04±2.39）％、（68.94±11.36）％和（74.47±16.37）％，BiTE组及TriTE组均明显高于PBS组（*P*值均<0.01），而BiTE组及TriTE组之间差异无统计学意义（图3B），表明CD33-BiTE及CD33-TriTE组均可刺激T细胞活化。培养第8天检测CD8^+^细胞表达比例，PBS组、BiTE组、TriTE组CD8^+^ T细胞比例分别为（32.74±4.19）％、（74.32±5.12）％及（80.35±3.79）％，BiTE组及TriTE组均明显高于PBS组（*P*值均<0.01），而BiTE组及TriTE组之间差异无统计学意义（图3C），表明CD33-BiTE及CD33-TriTE可使T细胞向CD8^+^ T细胞转化。

**图3:**
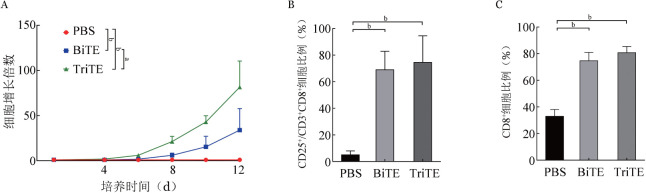
CD33-BiTE及CD33-TriTE对T细胞增殖（A）、活化（B）及分群（C）的影响（^a^ *P*<0.05、^b^ *P*<0.01） BiTE：双特异性T细胞衔接器；TriTE：三特异性T细胞衔接器

3. BiTE及TriTE对T细胞杀伤功能影响：将T细胞与表达CD33的Molm13细胞系按2∶1效靶比共培养，同时加入0.1 nmol/L CD33-BiTE及CD33-TriTE进行T细胞脱颗粒实验。CD33-BiTE及CD33-TriTE在表达CD33的Molm-13细胞中，PBS组、BiTE组及TriTE组CD8^+^ T细胞中CD107a表达比例分别为（10.73±4.85）％、（32.68±9.85）％及（34.96±11.95）％（图4A），其中PBS组明显低于BiTE组和TriTE组（*P*值均<0.01），而BiTE组与TriTE组差异无统计学意义。将不同浓度（0.001、0.01、0.1、1 nmol/L）BiTE及TriTE加入T细胞及Molm13-Luc2的共培养体系（效靶比为2∶1）中，共培养72 h，根据特异性杀伤比例计算浓度杀伤曲线，两组特异性杀伤均与浓度相关，且两组促杀伤效果无明显差异（图4B）。根据浓度梯度杀伤曲线，BiTE和TriTE均在0.1 nmol/L时达到杀伤峰值，故选用0.1 nmol/L 进行后续实验。将0.1 nmol/L CD33-BiTE及CD33-TriTE分别与表达CD33的Molm13细胞系及不表达CD33的Namalwa细胞系共培养，检测24 h及60 h的剩余靶细胞比例。在Molm13细胞系中，BiTE组与TriTE组均较PBS组杀伤明显（*P*值均<0.05），而Namalwa细胞系中，BiTE组杀伤作用较PBS组明显（*P*<0.05），而TriTE组与PBS组差异无统计学意义。

**图4:**
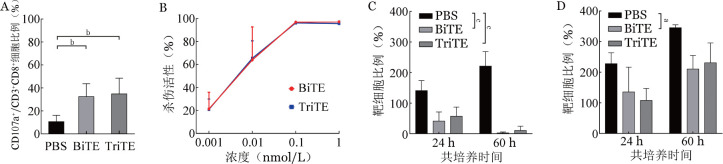
CD33-BiTE及CD33-TriTE介导T细胞与靶细胞共培养脱颗粒实验及杀伤实验（^a^*P*<0.05，^b^*P*<0.01，^c^*P*<0.0001） BiTE：双特异性T细胞衔接器；TriTE：三特异性T细胞衔接器；A：脱颗粒实验；B：浓度梯度杀伤曲线；C：对Molm13细胞杀伤曲线；D：对Namalwa细胞杀伤曲线

4. BiTE及TriTE对T细胞免疫检查点的影响：将CD33-BiTE与CD33-TriTE加入Molm13细胞系与T细胞共培养24 h后，检测剩余靶细胞中PD-L1的表达情况。结果显示PBS组、BiTE组及TriTE组靶细胞PD-L1阳性率分别为（13.33±3.34）％、（46.32±9.77）％及（18.87±5.74）％，其中BiTE组明显高于PBS组及TriTE组（*P*值均<0.05），而TriTE组与PBS组差异无统计学意义（图5A）。表明与CD33-TriTE相比，CD33-BiTE在杀伤的同时更容易诱导肿瘤细胞系表达PD-L1。共培养36 h后，PBS组、BiTE组及TriTE组CD8^+^ T细胞PD-1阳性率分别为（0.10±0.09）％、（17.80±3.80）％及（11.26±2.87）％，BiTE组和TriTE组较PBS组均明显升高（图5B）。构建过表达PD-L1的Molm13细胞系与T细胞共培养72 h，在0.1 nmol/L浓度下，TriTE组的T细胞对过表达PD-L1的Molm13细胞系杀伤能力更强（*P*<0.05）（图5C），提示可能在肿瘤细胞出现PD-L1表达的免疫抑制状态下，CD33-TriTE对肿瘤细胞的杀伤较CD33-BiTE更好。

**图5:**
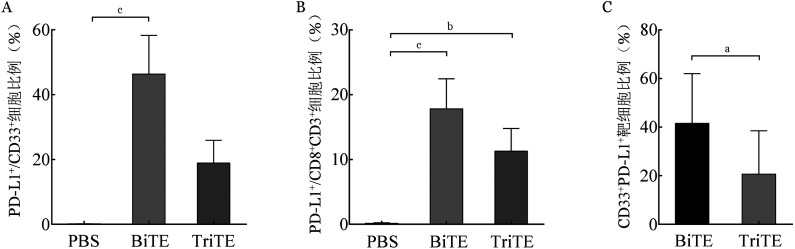
CD33-BiTE及CD33-TriTE诱导共培养的Molm13细胞系PD-L1表达（A）、T细胞PD-1表达（B）及对Molm13-PDL1细胞系杀伤实验（C） BiTE：双特异性T细胞衔接器；TriTE：三特异性T细胞衔接器

## 讨论

AML的传统化疗及造血干细胞移植后，仍有部分对化疗不敏感的难治性患者及复发患者，这部分患者预后较差。大多数AML肿瘤细胞表达CD33，而且在肿瘤干细胞上仍有表达[9]，因此提供了靶向CD33的各种免疫治疗的可能。目前靶向CD33治疗急性髓系白血病的免疫治疗多种多样，如嵌合抗原受体T细胞疗法[10]–[11]及抗体偶联药物[12]–[13]等。

特异性T细胞衔接器的理论基础在于一端为CD3抗体连接并活化T细胞，而另一端为肿瘤靶细胞抗体连接肿瘤细胞，将肿瘤细胞与T细胞连接后可增加T细胞对肿瘤细胞的杀伤[14]。我们研究开发了一种靶向CD33的BiTE，并在BiTE的基础上加入CD80胞外段，构成TriTE。蛋白在体内的半衰期的长短与蛋白分子量大小有关[15]，当蛋白分子量小于70×10^3^时，直接从正常的肾小球中滤过，半衰期较短，而加入了CD80胞外段的TriTE的分子量大于70×10^3^，因此相对于BiTE而言，TriTE不会直接从肾小球滤过，因此可能会延长药物在体内的半衰期。同时CD80胞外段提供了共刺激信号，增加了T细胞增殖的活性。本实验室前期构建CD19的三特异性抗体可使T细胞增殖活化及促进其杀伤功能[16]。

有研究发现在进行BiTE治疗过程中，肿瘤细胞的PD-L1表达呈现升高趋势[17]，而PD-L1与肿瘤免疫逃逸相关[18]。在AML患者中，PD-L1表达增高与不良预后有关[19]。有研究报道，同样是靶向CD33-BiTE的AMG 330，在添加到原代AML细胞中引起PD-L1的上调。PD-L1的上调与细胞因子相关，其中IFN-γ和TNF-α诱导了PD-L1的表达[20]。与本实验CD33-BiTE能增加PD-L1的表达一致，而增加了CD80胞外段的CD33-TriTE却较少诱导肿瘤细胞PD-L1的表达。

目前研究发现，抗原提呈细胞上大量表达CD80时，PD-L1不能与PD-1结合，从而抑制T细胞激活。在蛋白水平上，也观察到了PD-1和CD80之间对PD-L1结合的竞争。因此，该研究团队认为可以使用可溶性CD80作为提高T细胞对肿瘤杀伤的药物[21]。另一方面，研究者发现在AMG 330的基础上加入PD-L1/PD-1阻滞剂能增强AMG 330对PD-L1升高的原代AML细胞的杀伤作用[20]。在此基础上，研究人员使用PD-L1胞外段连接靶向CD33-BiTE构建的CiTE，通过阻断PD-L1/PD-1结合，对高表达PD-L1的靶细胞具有更好的杀伤作用[6]。基于这些前期研究基础，我们使用CD80胞外段作为另一种阻断PD-1/PD-L1轴的方式，同样获得了对高表达PD-L1靶细胞的杀伤效果。因此我们推测CD33-TriTE可能对于PD-L1表达升高的AML患者更有效。

本实验研究BiTE和TriTE介导的特异性杀伤作用时，使用的T细胞为供者来源的，而靶细胞是细胞系，可能存在HLA不相合的情况，最好应使用同一患者来源的T细胞及肿瘤细胞重复杀伤实验。另外，BiTE与TriTE在体内对T细胞增殖和杀伤作用仍需进一步实验研究。综上，本研究中我们成功构建了双特异性及三特异性T细胞衔接器，验证了其对T细胞的增殖活化及促杀伤功能。通过比较发现，CD33-TriTE较CD33-BiTE增殖效果更强，且对PD-L1高表达的细胞杀伤作用更好。

## References

[1] Döhner H, Estey E, Grimwade D (2017). Diagnosis and management of AML in adults: 2017 ELN recommendations from an international expert panel[J]. Blood.

[2] Thol F, Ganser A (2020). Treatment of Relapsed Acute Myeloid Leukemia[J]. Curr Treat Options Oncol.

[3] 王 珍珍, 卢 杨, 徐 颖茜 (2020). 一种新的CD123嵌合抗原受体T细胞的构建及其抗白血病作用探究[J]. 中华血液学杂志.

[4] Baron J, Wang ES (2018). Gemtuzumab ozogamicin for the treatment of acute myeloid leukemia[J]. Expert Rev Clin Pharmacol.

[5] Nair-Gupta P, Diem M, Reeves D (2020). A novel C2 domain binding CD33xCD3 bispecific antibody with potent T-cell redirection activity against acute myeloid leukemia[J]. Blood Adv.

[6] Herrmann M, Krupka C, Deiser K (2018). Bifunctional PD-1× αCD3 × αCD33 fusion protein reverses adaptive immune escape in acute myeloid leukemia[J]. Blood.

[7] Sarhan D, Brandt L, Felices M (2018). 161533 TriKE stimulates NK-cell function to overcome myeloid-derived suppressor cells in MDS[J]. Blood Adv.

[8] Pérez-Oliva AB, Martínez-Esparza M, Vicente-Fernández JJ (2011). Epitope mapping, expression and post-translational modifications of two isoforms of CD33 (CD33M and CD33m) on lymphoid and myeloid human cells[J]. Glycobiology.

[9] Hauswirth AW, Florian S, Printz D (2007). Expression of the target receptor CD33 in CD34+/CD38-/CD123+ AML stem cells[J]. Eur J Clin Invest.

[10] Li S, Tao Z, Xu Y (2018). CD33-Specific Chimeric Antigen Receptor T Cells with Different Co-Stimulators Showed Potent Anti-Leukemia Efficacy and Different Phenotype[J]. Hum Gene Ther.

[11] Kim MY, Yu KR, Kenderian SS (2018). Genetic Inactivation of CD33 in Hematopoietic Stem Cells to Enable CAR T Cell Immunotherapy for Acute Myeloid Leukemia[J]. Cell.

[12] Han YC, Kahler J, Piché-Nicholas N (2021). Development of Highly Optimized Antibody-Drug Conjugates against CD33 and CD123 for Acute Myeloid Leukemia[J]. Clin Cancer Res.

[13] Kovtun Y, Noordhuis P, Whiteman KR (2018). IMGN779, a Novel CD33-Targeting Antibody-Drug Conjugate with DNA-Alkylating Activity, Exhibits Potent Antitumor Activity in Models of AML[J]. Mol Cancer Ther.

[14] Goebeler ME, Bargou R (2016). Blinatumomab: a CD19/CD3 bispecific T cell engager (BiTE) with unique anti-tumor efficacy[J]. Leuk Lymphoma.

[15] Strohl WR (2015). Fusion Proteins for Half-Life Extension of Biologics as a Strategy to Make Biobetters[J]. BioDrugs.

[16] 陈 曼玲, 彭 楠, 刘 晓雨 (2021). 一种新的靶向CD19抗原的三特异性T细胞衔接器的制备及其抗白血病作用研究[J]. 中华血液学杂志.

[17] Köhnke T, Krupka C, Tischer J (2015). Increase of PD-L1 expressing B-precursor ALL cells in a patient resistant to the CD19/CD3-bispecific T cell engager antibody blinatumomab[J]. J Hematol Oncol.

[18] Jiang X, Wang J, Deng X (2019). Role of the tumor microenvironment in PD-L1/PD-1-mediated tumor immune escape[J]. Mol Cancer.

[19] Chen C, Liang C, Wang S (2020). Expression patterns of immune checkpoints in acute myeloid leukemia[J]. J Hematol Oncol.

[20] Krupka C, Kufer P, Kischel R (2016). Blockade of the PD-1/PD-L1 axis augments lysis of AML cells by the CD33/CD3 BiTE antibody construct AMG 330: reversing a T-cell-induced immune escape mechanism[J]. Leukemia.

[21] Sugiura D, Maruhashi T, Okazaki IM (2019). Restriction of PD-1 function by cis-PD-L1/CD80 interactions is required for optimal T cell responses[J]. Science.

